# Prevalence and Impact of Hepatitis on the Quality of Life of Patients

**DOI:** 10.5005/jp-journals-10018-1142

**Published:** 2016-07-09

**Authors:** Pardeep Mittal, Prithpal S Matreja, HK Rao, PML Khanna

**Affiliations:** 1 Gian Sagar Medical College and Hospital, Patiala, Punjab, India; 2 Department of Pharmacology, Gian Sagar Medical College and Hospital, Patiala, Punjab, India; 3 Department of Internal Medicine, Gian Sagar Medical College and Hospital, Patiala, Punjab, India

**Keywords:** Females, Health, Hepatitis, Males, Quality of life.

## Abstract

**Background:**

Hepatitis is a disorder which is emerging as major health problem with increasing morbidity and mortality. Inappropriate management of this disease leads to several complications that can impair the health related quality of life (HRQoL) of the individuals. There, we designed this study to assess the HRQoL in patients suffering from hepatitis.

**Materials and methods:**

This prospective, observational study was conducted for 2 months in patients with hepatitis. All patients with hepatitis were recruited in the study after giving written informed consent. The participants were given both World Health Organization Quality of Life-Bref (WHO QoL-Bref) and short form (SF-36) health survey questionnaires for assessing the quality of life (QoL) of patients. The participants were given counseling of lifestyle modification and underwent a thorough medical examination and a detailed history was taken.

**Results:**

A total of 65 volunteers participated in the study, out of which 30 were control and 35 were patients suffering from hepatitis; hepatitis C was found to be more prevalent. The patients with hepatitis had a significantly poor QoL as compared to control. In hepatitis patients, physical health and physical functioning was better in males as compared to females. Social relationships were also good in males than females. But females had higher score in role limitation due to physical health, emotional wellbeing, social functioning, pain, general health as per SF-36 scores but it was not statistically significant.

**Conclusion:**

Hepatitis affected the HRQoL in Indian population and parameters seem to be heterogeneously affected in males and females.

**How to cite this article:**

Mittal P, Matreja PS, Rao HK, Khanna PML. Prevalence and Impact of Hepatitis on the Quality of Life of Patients. Euroasian J Hepato-Gastroenterol 2015;5(2):90-94.

## INTRODUCTION

Hepatitis is an inflammation of the liver, most commonly caused by a viral infection. There are five main hepatitis viruses, referred to as types A, B, C, D and E.^1^ In India, hepatitis E virus (HEV) infection is responsible for 30 to 70% of cases of acute sporadic hepatitis and is the major cause of acute liver failure (ALF).^2^ About 15 to 30% of acute hepatitis in India is due to hepatitis B virus (HBV).^3^ About 50% of chronic liver disease (CLD) is due to HBV and 20% is due to hepatitis C virus (HCV) infection.^4^ Nearly 119,000 cases of all-cause viral hepatitis were reported in India in 2012. The Integrated Disease Surveillance Programme of the National Center for Disease Control (NCDC) received notification of 290,000 cases of acute viral hepatitis in 2013.^5^ Globally, HBV and HCV together are estimated to have led to 500 million chronically infected persons and one million deaths annually.^6,7^ In viral hepatitis, the presence of the virus in the liver cells causes the immune system to attack the liver, resulting in inflammation and impaired function.^8^ Acute infection may occur with limited or no symptoms, or may include symptoms, such as jaundice, dark urine, extreme fatigue, nausea, vomiting, abdominal pain.^1^ Previous studies have demonstrated a significant decreased quality of life (QoL) in patients suffering from hepatitis.^9^ Chronic hepatitis B and compensated cirrhosis have a moderate impact on health-related quality of life (HRQoL), and there is a large detrimental effect on QoL associated with decompensated cirrhosis and hepatocellular carcinoma.^10^ Hepatitis C virus infection significantly reduces HRQoL, even in the absence of cirrhosis, and that successful treatment of HCV is associated with an improvement in HRQoL.^11^ Hepatitis C is commonly accompanied by fatigue and depression, followed by a decreased interest in sex. Additionally, antiviral medications typically used to battle hepatitis C may cause sexual dysfunction and decreased libido. Sexual dysfunction is the most frequently encountered side effect of many antidepressant medications used to treat the depression and anxiety associated with combination treatment for HCV.^12^ A study (living with hepatitis C and treatment: the personal experiences of patients) done in Australia revealed that chronic hepatitis C and combination therapy had an enormous impact on the lives of the patients, their partners and families. The illness and treatment had significant physiological, sexual effects that had an impact on QoL; however, the social and psychological consequences of living with a highly stigmatized disease with an unknown course and outcome cannot be underestimated.^13^ There are limited numbers of studies done in this field in India, hence we designed this to study the prevalence and impact of hepatitis on the QoL of patients in India.

## MATERIALS AND METHODS

This prospective, cross-sectional study was conducted in collaboration of Department of Medicine and Pharmacology, Gian Sagar Medical College and Hospital, Patiala, Punjab, India, for 2 months from April 2014 to May 2014 in patients visiting the out-patient department (OPD) with hepatitis. The study was approved by Institutional Ethics Committee and a valid informed consent was taken from the subjects before enrolment into the study. The patients of either sex in the age group of 18 to 35 years of age, diagnosed with viral hepatitis and who gave their written informed consent were included in the study. All patients with chronic medical, surgical conditions, with organic brain syndrome, chronic mental illness, non-cooperative and unwilling patients were excluded from the study. All the patients visiting the OPD of medicine and suffering from hepatitis underwent a through medical examination and then the severity of hepatitis was determined. Control group was taken from subjects who were visiting the OPD and not suffering from hepatitis. Patients who fulfilled the inclusion and exclusion criteria were enrolled in the study if they gave written informed consent. Patients were assessed for quality of life parameters by the researcher.

## OUTCOME MEASURES

### Short Form Health Survey (SF-36)

The questionnaire contains 36 items integrated in multi-item scales measuring eight generic health concepts: physical functioning (PF), social functioning (SF), role physical (RP), bodily pain (BP), mental health (MH), role emotional (RE), vitality (VT), and general health (GH). Scoring included transformation of raw scores for each subscale to a 0 to 100 scale and a higher scores representing better QoL.^14^

The World Health Organization Quality of Life-Bref (WHO QoL-Bref): was monitored at visit. This is a 26-item self-administered generic questionnaire, a short version of WHO QoL-100 scale. It can be analyzed from perspective of either six domains (physical health, psychological health, level of independence, social relationships, environment, and spiritual) or four domains (physical health, psychological health, social relations and environment).^15^

Four domains are defined for WHO QoL-Bref, based on its 26 items: domain 1, physical health, is on activities of daily living, dependence on medicinal substances and medical aids, energy and fatigue, mobility, pain and discomfort, sleep and rest, and work capacity. Domain 2, psychological health, includes bodily image and appearance, negative feelings, positive feelings, self-esteem, spirituality, religion, personal beliefs, thinking, learning, memory and concentration. Domain 3, social relationships, covers personal relationships, social support, and sexual activity. Domain 4, environment, assesses financial resources, freedom, physical safety and security, health and social care (accessibility and quality), home environment, opportunities for acquiring new information and skills, participation in and opportunities for recreation and leisure activities, physical environment (pollution, noise, traffic and climate), and transport. The raw score of each domain was then transferred to standardized score of 0 to 100, in order to maintain uniformity in scores. Higher scores mean better QoL of patients. The QoL index of each domain and their associations with demographic factors were assessed.^16-18^

## STATISTICAL ANALYSIS

The data were tabulated as mean ± standard deviation (SD). Results were analyzed using nonparametric tests (Chi-square test), parametric tests (two tailed student t-test) and correlation (Pearson correlation coefficients) analysis. A p < 0.05 was considered statistically significant.

## RESULTS

A total of 65 participants were enrolled in the study. All the patients gave informed consent and were included in the analysis of result. A total of 30 participants were control and 35 patients with hepatitis. In the control group, there were 11 females and 19 males, the mean age in control group was 32.47 ± 2.45 years. In 35 patients with hepatitis, four patients were of hepatitis B and 31 patients were of hepatitis C. This shows the prevalence of HCV in the study area. The mean age of participants with hepatitis was 31.63 ± 3.15 years, a total of nine males and 26 females were enrolled in the study.

## SF-36 SCORES

The baseline SF-36 scores are shown in Table 1 for all the participants. The control group had better scores for all parameters. There was a significant (p < 0.05) compromise in the component of energy/fatigue, social functioning, pain and general health as is evident by low scores in all these components in patients suffering from hepatitis.

**Graph 1:**
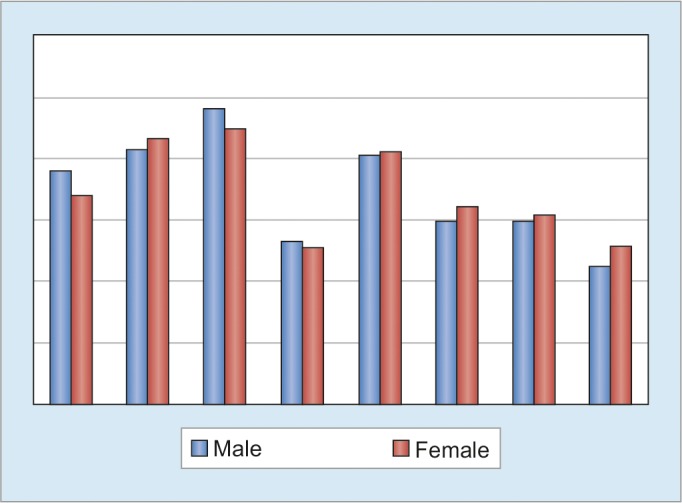
Short Form-36 (SF-36) scores in both groups (males *vs* females)

The hepatitis patients were divided into two groups based on their gender (males *vs* females). The SF-36 scores in both groups are shown in Graph 1. Scoring included transformation of raw scores for each subscale to a 0 to 100 scale and a higher scores representing better QoL. There were significantly good physical functioning in males (76.11 ± 16.54 *vs* 67.89 ± 13.80) as compared to females. The males also had more, though statistically not significant (p > 0.05) role limitation due to emotional problem (96.30 ± 11.11 *vs* 89.74 ± 15.69), energy/fatigue (53.33 ± 7.5 *vs* 51.34 ± 8.67) as compared to females. On the other hand, females have higher role limitation due to physical health (83.33 ± 21.65 *vs* 86.54 ± 17.65), emotional wellbeing (81.33 ± 6.33 *vs* 82.46 ± 3.77), social functioning (59.72 ± 10.42 *vs* 64.42 ± 13.08), pain (59.44 ± 9.34 *vs* 62.02 ± 10.72), general health (45 ± 11.18 *vs* 51.73 ± 8.36). None of the parameters had statistical significance.

## WHO QOL-BREF SCORES

The baseline WHO QoL-Bref scores are shown in Table 2 for all the patients. There was a significant (p < 0.05) compromise in all the domains, i.e. physical, psychological, social relationship and environment as is evident by low scores in all these components in patients suffering from hepatitis.

**Graph 2:**
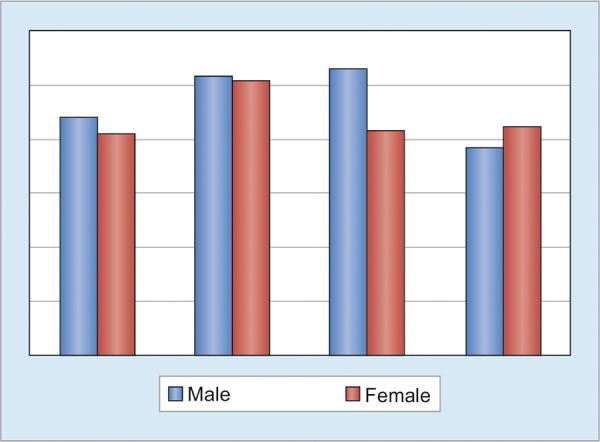
World Health Organization Quality of Life-Bref scores in both groups (males *vs* females)

The hepatitis patients were divided into two groups based on their gender (males *vs* females). World Health Organization Quality of life-Bref scores are shown in Graph 2. The higher scores mean better QoL of patients. Male had higher scores in domain 1, that is, physical health (43.89 ± 8.67 *vs* 41.23 ± 7.74), almost equal in domain 2, i.e. psychological health (51.56 ± 11.51 *vs* 51 ± 14.40), higher in domain 3, i.e. social relationship (52.89 ± 26.37 *vs* 41.84 ± 21.02) but lesser in domain 4, i.e. environment (38.22 ± 12.66 *vs* 42.69 ± 8.19) but it was not statistically significant.

**Table 1:** **Table 1:** Baseline SF-36 scores in all the participants

*Parameter*		*Hepatitis* *(n = 35)* *(Mean ± SD)*		*Control* *(n = 30)* *(Mean ± SD)*		*p-value*	
Physical functioning		70 ± 14.75		86.5 ± 9.48		<0.05*	
Role limitations due to		85.71 ± 18.48		92.5 ± 13.38		>0.05	
physical health							
Role limitations due to		91.43 ± 14.78		93.3 ± 13.56		>0.05	
emotional problem							
Energy/fatigue		51.86 ± 8.32		78.3 ± 8.02		<0.05*	
Emotional well being		82.17 ± 4.48		87.2 ± 5.29		>0.05	
Social functioning		63.21 ± 12.48		80.42 ± 11.22		<0.05*	
Pain		61.36 ± 10.31		74.08 ± 4.93		<0.05*	
General health		50 ± 9.47		71.67 ± 9.41		<0.05*	

*p < 0.05; significant as compared to patients with hepatitis

**Table 2:** **Table 2: **Baseline WHO QoL-Bref scores (0-100) in all the participants

		*0-100*					
*Domains*		*Hepatitis* *(n = 35)* *(Mean ± SD)*		*Control* *(n = 30)* *(Mean ± SD)*		*p-value*	
Domain 1/physical		41.91 ± 7.95		67.67 ± 14.18		<0.05*	
Domain 2/psychological		51.14 ± 13.55		65.53 ± 8.86		<0.05*	
Domain 3/social		44.69 ± 22.64		73.50 ± 20.06		<0.05*	
Relationship							
Domain 4/environment		41.54 ± 9.53		62.07 ± 15.35		<0.05*	

*p < 0.05; significant as compared to patients with hepatitis

### Correlation

Estimates of correlation for SF-36 scores with WHO QoL-Bref Scores was observed and it was seen that SF-36 Score had no statistically significant (p > 0.05) correlation with physical health, psychological, and social relationship in both groups.

## DISCUSSION

Hepatitis is a disorder which is emerging as major health problems with increasing morbidity and mortality. Also the recent studies show significant decrease in the QoL of patients. The present study was undertaken to assess the health related QoL in patients suffering from hepatitis. As compared to control group the patients with hepatitis had significantly compromised QoL. The QoL was impaired in both males and females as evident by low scores in both SF-36 and WHO QoL-Bref scores. The psychological impact in more than emotional impact on the QoL of patients when scores were compared from 0 to 100 between the two scales. Physical health and physical functioning is better in males as compared to females as per WHO QoL-Bref Scores and SF-36 respectively. Also the males had significantly higher score in domain 3, i.e. social relationship as per WHO QoL-Bref scores. But the females have higher score in role limitation due to physical health, emotional well being, social functioning, pain, general health as per SF-36 scores.

In a cross-sectional population-based study done in Brazil by using WHO QoL-Bref scale in 108 hepatitis C patients shows the lowest score for the social relationships domain and the highest score for the environment domain.^19^ The results of this study are similar to our study in the aspect that our study also shows decreased score for social relationships. The difference in this study and our study is that our study shows decreased score for other three domains also.

Another article focusing on the QoL between males and females shows those females shows worse QoL than males, supporting that gender differences in hepatitis are also important when assessing QoL.^20^ The results of this study are similar to our study in aspect that our study also shows decrease in physical health and physical functioning of females as compared to males. The difference in our study with this study is that our study shows females has higher score in role limitation due to physical health, emotional wellbeing, social functioning, pain, general health as per SF-36 scores.

There are certain limitation in our study firstly the sample size could have been larger but, the duration of study was only 2 months, hence we tried to include patients who fulfilled the eligibility criteria. Secondly, a comparison with the intervention arm could be done, but any intervention could have prolonged the duration of study and we would not have been able to complete the study in the allotted 2 months.
